# Factors Associated with Anemia Status Among Children Aged 6–59 months in Ghana, 2003–2014

**DOI:** 10.1007/s10995-019-02865-7

**Published:** 2020-02-06

**Authors:** Luke M. Shenton, Andrew D. Jones, Mark L. Wilson

**Affiliations:** 1 Department of Epidemiology, School of Public Health, University of Michigan, 1415 Washington Heights, Ann Arbor, MI 48109 USA; 2 Department of Nutritional Sciences, School of Public Health, University of Michigan, 1415 Washington Heights, Ann Arbor, MI 48109 USA

**Keywords:** Anemia disease risk, West Africa, Childhood health disparities, Demographic and health survey (DHS), Maternal and child nutrition

## Abstract

**Background:**

In 2008, 78% of Ghanaian children under 5 years old were anemic versus 48% of children globally. In this study, we identified proximal and distal determinants of severe–moderate anemia and mild anemia related to socioeconomic status, nutrition, and health access.

**Methods:**

Using data from the 2003, 2008, and 2014 Ghana Demographic and Health Surveys (GDHS), the odds of severe–moderate anemia and mild anemia compared to no anemia, in relation to various hypothesized risk factors, were assessed using a multivariable, multinomial logistic regression, which accounted for survey design.

**Results:**

From among 7739 households sampled, severe–moderate anemia was found in approximately 52%, 56%, and 40% of children during 2003, 2008, and 2014, respectively. The proportion of children diagnosed as not anemic was 26% in 2003, 22% in 2008, and 33% in 2014. There are large disparities in anemia prevalence among regions and between urban and rural areas. In 2014, children who were younger (aged 6–24 months), had a recent fever, were from poorer families, and whose mothers were less educated had greater odds of being severely–moderately anemic. These results remained significant when controlling for other risk factors. Predictors of anemia in Ghana remained relatively consistent among the three time periods when the GDHS was administered.

**Conclusions:**

The prevalence of anemia in Ghana among children aged 6–59 months has remained unacceptably high. To reduce anemia in these children, the Ghanaian government should concentrate on educating women through both the traditional school system and antenatal care visits.

## Significance

*What is Already Known on This Subject?* Existing literature using GDHS data shows there are child, household, and sociocultural factors associated with anemia in Ghana. *What This Study Adds?* This is the first article, that we know of, to assess predictors of anemia in Ghana over three rounds of GDHS data. Younger child age, fever in the last 2 weeks, lower household wealth, less maternal education, and maternal anemia were significantly associated with severe–moderate anemia among children in 2014. The predictors remained relatively constant over time, with similar risk factors being seen in 2003 and 2008. These results can inform policy makers in Ghana.

## Introduction

Globally, undernutrition is one of the largest contributors to child mortality, causing an estimated 45% of total deaths of children younger than five years (Black et al. 2013). Undernutrition encompasses a broad range of public health problems, including deficiencies of micronutrients, especially iron (WHO 2008a), that often contribute to anemia. Anemia is of particular concern in children because it can cause deficits in cognitive and behavioral development and function (Beard and Connor 2003; McCann and Ames 2007). Understanding the complex etiologies of various types of anemia is of particular importance, due to the high prevalence of anemia in many parts of the world. One 2011 estimate indicated that 43% of children under 5 years old had anemia worldwide, with prevalence as high as 71% in parts of central and western Africa (Stevens et al. 2013).

Because of its large contribution to morbidity and mortality, anemia remains an important public health problem, especially in sub-Saharan Africa. In 2008, 78% of Ghanaian under-five children were anemic, with 56% of these children suffering from severe or moderate anemia (Ewusie et al. 2014). Due to this high prevalence, improved understanding of associated factors is needed to better tailor interventions for reducing the burden of anemia in Ghana.

**Fig. 1:**
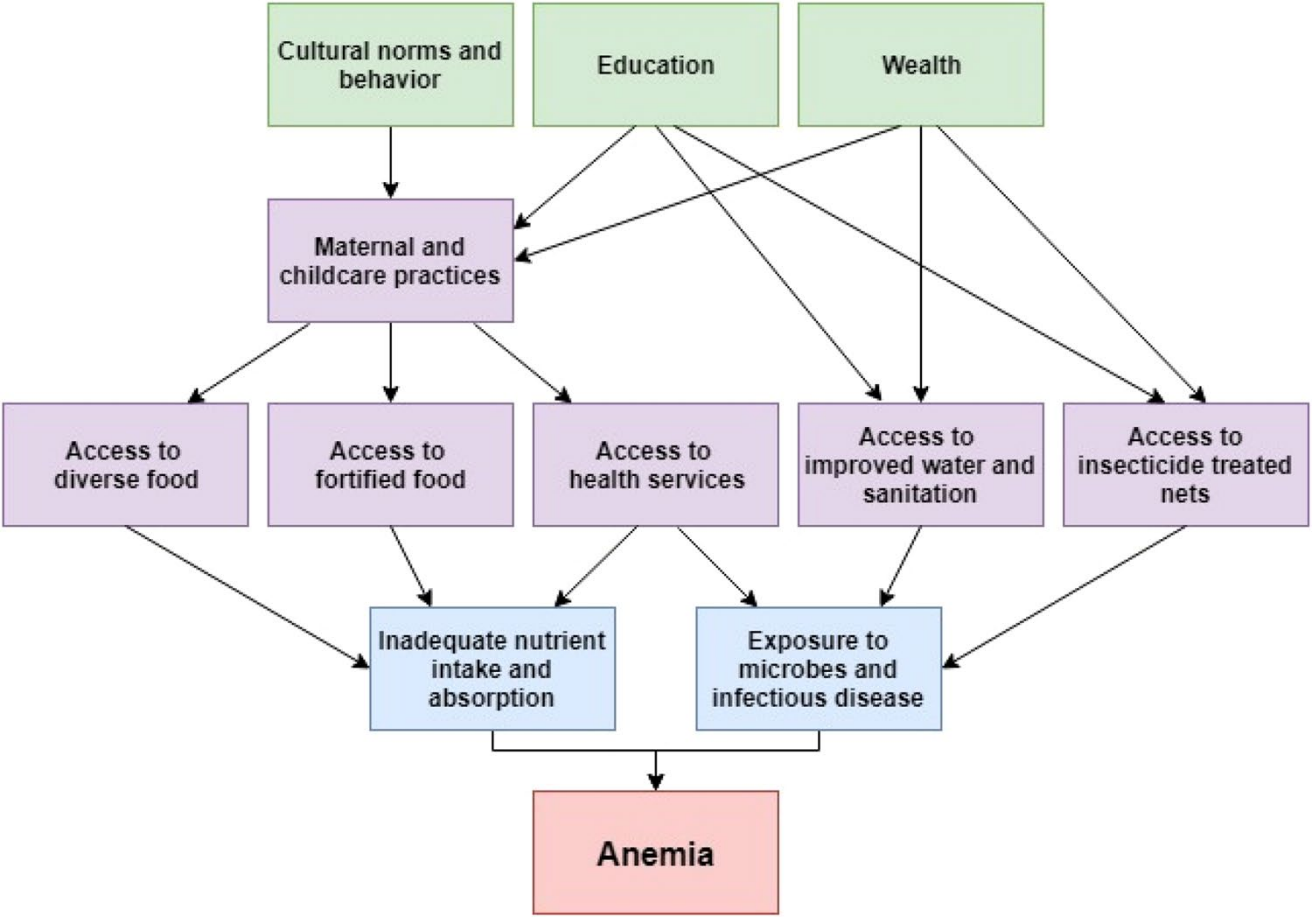
Theoretical framework for predictors of anemia

On a biological level, anemia is usually caused by decreased erythrocyte production or increased erythrocyte loss (Camaschella 2015). In addition to such biological causes, there are many complex, upstream environmental, behavioral and social factors that contribute to risk for anemia, especially in children (Balarajan et al. 2011; Siekmans et al. 2014). These factors are often divided into three major categories. First, there are immediate causes, which include inadequate nutrient and absorption (i.e. nutrient supplementation) and exposure to infectious disease (i.e. fever, malaria, intestinal parasites) (Mueller et al. 2017; Paganini and Zimmermann 2017). Secondly, there are underlying causes at the household/family level, such as access to water and sanitation, availability of health services, childcare practice (e.g. breastfeeding) access to diverse food sources, use of insecticide-treated bednets (ITNs), and knowledge about anemia prevention (Larsen et al. 2017). Lastly, the most distal causes are related to the larger sociocultural context, such as education, wealth, and cultural norms and behavior (Ngnie-Teta et al. 2009). Figure 1 provides a graphical depiction of these hypothesized relationships and more information can be found in the methods section. An updated analysis of the relationship between such risk factors and anemia in a nationally representative sample of Ghanaian children is needed given that most existing studies are limited in some way. Investigations into anemia in Ghana generally have involved small sample sizes, and only evaluated risk within small areas (Egbi et al. 2014; Klinkenberg et al. 2006; VanBuskirk et al. 2014). Other studies that used a nationally representative sample are limited by only considering prevalence estimates for anemia or a sample subset of potential predictors (Ewusie et al. 2014; Saaka and Galaa 2017).

The objective of our study, therefore, was to determine the prevalence and predictors of anemia among Ghanaian children aged 6–59 months using nationally representative samples from the Ghana Demographic and Health Survey (GDHS), covering 2003, 2008, and 2014. Thereby, we undertook a more in-depth analyses of anemia risk that also evaluated whether and how there may have been changes in anemia prevalence and correlates over time. We hypothesized many previously identified predictors of childhood anemia, such as malaria and diarrheal disease, would be associated with anemia, as would maternal factors including education and maternal autonomy (Balarajan et al. 2011; Siekmans et al. 2014). In addition, we hypothesized there would be decreasing anemia prevalence over time commensurate with improvements in equity across various socioeconomic and demographic groups.

## Methods

### Study Population

This study analyzed Ghana DHS (GDHS) data for 2003, 2008, and 2014. All three surveys and supporting documentation are available from reports published on DHS Program website (Ghana Statistical Service, Ghana Health Service and ICF International 2009, 2015; Ghana Statistical Service, Noguchi Memorial Institute for Medical Research and ORC Macro 2004; United States Agency for International Development 2017). The three surveys used different phases of the DHS; phase IV in 2003, phase V in 2008, and phase VII in 2014. However, the sampling scheme was virtually identical across these phases, so only the 2014 methods are explained here. The 2014 GDHS used the Ghana 2010 Population and Housing Census as the sampling frame, which consists of 37,641 enumeration areas (EAs) that cover the entire country. The 2008 and 2003 GDHS surveys used the 2000 Population and Housing Census; all regions of Ghana showed population growth between the 2000 and the 2010 census (Ghana Statistical Service 2012). EAs are geographic areas that serve as counting units for the Ghanaian governmental census and contained an average of 145 households in the 2010 census.

The GDHS followed a stratified, two-stage, sample design, which allows estimation of key indicators at the national level, in both urban and rural areas, and in each of Ghana’s ten regions. In the first stage, clusters were selected, which consisted of EAs; 216 clusters from urban areas and 211 from rural areas, for a total of 427 clusters.

In the second stage, approximately 30 households were selected from each cluster to yield a total sample size of 12,831 households. The sample design selected an approximately equal sample size in each region; therefore, the sample is not self-weighting at the national level, so weights were calculated such that results are also representative at the national level.

Information about the household, reproductive health, maternal care, childhood immunization, and childcare was collected via in-person interviews with women aged 15–49 years. The population used for analysis was the children from each household aged 6–59 months for whom anemia data are available.

### Derived Variables

Our outcome of interest was anemia status in 6–59 month old children, which we grouped into three levels based on Hb concentration: severe–moderate anemia (< 100 g/L), mild anemia (100–109 g/L), or non-anemic (≥ 110 g/L) (WHO 2011). We combined severe and moderate anemia into one category because a very small number of children had severe anemia giving the category very low statistical power for analysis. Blood samples were taken via a finger prick (or heel prick for children age 6–11 months) and collected in a microcuvette. A HemoCue analyzer was used to measure Hb concentration. Hb levels were adjusted for altitude.

The explanatory variables we analyzed were based on various conceptual models of anemia determinants, plus variables found to be important predictors of anemia in previous studies (Balarajan et al. 2011; Saaka and Galaa 2017; Siekmans et al. 2014). These explanatory variables were related to sociodemographic characteristics and health access, and were examined for any relationship with the children's anemia status.

Immediate causes of anemia, which included recent diarrhea (last 2 weeks), recent fever (last 2 weeks), and recent treatment for parasitic worms (last 6 months), were dichotomized into yes or no answers. Underlying causes comprised most variables, and included age of the child’s mother, child’s sex, iron supplementation, childcare practice (e.g. breastfeeding), dietary diversity, environmental health (e.g. access to a toilet facility and clean water), access to ITNs, the number of children in the household, and health insurance coverage. Dietary diversity was characterized by the number of different food groups the child consumed in the previous 24 h. We reclassified the GDHS food data into seven distinct food groups: (i) grains, roots and tubers; (ii) legumes and nuts; (iii) dairy products; (iv) flesh foods (meats/fish/poultry); (v) eggs; (vi) vitamin A-rich fruits and vegetables; and (vii) other fruits and vegetables. These food groups underlie the WHO Minimum Dietary Diversity (MDD) indicator for children—an indicator validated against the micronutrient adequacy of child diets (Working Group on Infant and Young Child Feeding Indicators 2006; WHO 2008). The dietary diversity score, which ranged from zero to seven food groups, was then dichotomized to create the MDD that was met by consuming four or more food groups.

The more "distal" hypothesized causes of anemia included household wealth, maternal education, religion, ethnicity, and maternal autonomy. The household wealth index was derived by grouping survey participants into quintiles based on a standardized asset-based score. Measures of maternal autonomy included number of other wives, maternal decision-making autonomy, and maternal attitudes towards being beaten. For decision making autonomy, we classified mothers as having none, some, or full decision-making autonomy based on their answers to questions about decision making behaviors in their households. DHS surveyors asked mothers about who in their household makes decisions related to their own health-care, large household purchases, and visits to relatives. Mothers could answer that they alone make the decision, they jointly make the decision with their husband, their husband alone makes the decision, or someone else makes the decisions. If the mother played a role in all decisions she had full decision-making autonomy, if she played a role in making some decisions she had partial decision-making autonomy, and if she did not play a role in making decisions she had no decision-making autonomy. For attitudes towards being beaten, we dichotomized mothers’ responses to questions about wife beating into yes (beating by their husband is justifiable) or no (not justifiable). DHS surveyors asked mothers if they thought being beaten by their husband was justifiable in five scenarios: they leave the house without telling their husband, they neglect the children, they argue with their husband, they refuse to have sex, and they burn the food.

### Statistical Analysis

The distribution of factors are presented using descriptive statistics, and their unadjusted relationship with severe–moderate and mild anemia were assessed through a Rao-Scott chi-square test. A multivariable, multinomial, logistic regression model was developed to determine the associations between independent factors and anemia status. The regression models were built by considering previous studies, and included all factors other than dietary diversity and breastfeeding. Dietary diversity was not included due to a small overall sample size (N = 1373) and breastfeeding was excluded due to high collinearity with child’s age. For all other variables, observations with missing data were removed from the multivariable model. However, the removed observations accounted for only a small proportion of the total number of observations. Ethnicity, originally a nine-category variable, was collapsed into six categories, while maternal education was collapsed from six to three categories, in both cases due to small cell size, which was defined as under 150 observations. The 2003 regression model differed slightly from those of 2008 and 2014 due to missing variables such as iron supplementation, drugs for parasites, and health insurance status. All descriptive, bivariate, and multivariable analyses were conducted using appropriate clustering, stratification, and weighting statements to account for the complex sample design. The regression model generated odds ratios (OR) and 95% confidence intervals (CI). Variance was estimated through the Taylor series linearization method. Significance was assessed at an α level of 0.05, with all analyses being done in SAS version 9.4 (SAS Institute, Cary, NC, USA).

## Ethical Approval

This study was exempt from ethical approval because it was limited to publicly available datasets that contained no personally identifiable information. All participants provided informed consent to the GDHS interviewers before being enrolled into the study.

## Results

### Study Population

The 2003, 2008, and 2014 GDHS datasets provided information on 3183, 2168, and 2388 children aged 6–59 who had Hb measurements taken, respectively. The sociodemographic characteristics of these children are summarized in Table 1. In 2014, ~ 48% of children had slept under a mosquito net the previous night; a significant increase from 2003 when only 15% had. Around 12% of children had diarrhea in the previous 2 weeks, while 15% had a fever in the last 2 weeks, an improvement over previous years. A quarter of children in 2014 had no toilet facility compared to one-third in 2003 and 17% had access to flush toilet in 2014 compared to 7% in 2003. In 2014, 29% of mothers (11% less than in 2003) had completed no education, while only 10% of mothers had completed secondary education or higher. Approximately 58% of mothers had full decision-making autonomy in the household, while 7% had none; in contrast to the 26% and 40% of mothers with no and full decision-making autonomy, respectively in 2003. Across all survey years, the majority (75%) of children belonged to Christian households and were members of the Akan ethnic group (48%).

**Table 1:** Sociodemographic profile of children 6–59 months

Characteristics	2003		2008		2014	
	n	Weighted percentages (95% confidence intervals)	n	Weighted percentages (95% confidence intervals)	n	Weighted percentages (95% confidence intervals)
Anemia type	3183		2168		2388	
Severe–moderate		52.11 (49.95, 54.27)		55.81 (52.99, 58.63)		39.80 (36.78, 42.83)
Mild		22.02 (20.39, 23.64)		22.61 (20.54, 24.67)		27.00 (24.89, 29.11)
None		25.87 (23.98, 27.76)		21.58 (19.34, 23.83)		33.20 (30.18, 36.22)
Region	3183		2168		2388	
Western		10.13 (8.13, 12.12)		9.43 (7.48, 11.38)		10.59 (8.69, 12.50)
Central		8.86 (6.55, 11.16)		9.01 (7.01, 11.00)		11.69 (7.20, 16.18)
Greater Accra		10.73 (8.92, 12.54)		11.92 (9.83, 14.02)		15.62 (12.51, 18.73)
Volta		8.51 (7.47, 9.56)		8.74 (6.81, 10.68)		7.34 (5.67, 9.00)
Eastern		9.62 (8.22, 11.03)		8.62 (7.28, 9.96)		8.65 (7.10, 10.20)
Ashanti		17.71 (15.87, 19.55)		19.21 (16.60, 21.81)		17.36 (14.65, 20.06)
Brong Ahafo		11.25 (9.93, 12.56)		10.44 (8.71, 12.17)		9.60 (7.69, 11.51)
Northern		13.78 (11.97, 15.60)		14.97 (12.50, 17.43)		12.52 (9.63, 15.40)
Upper east		3.09 (2.43, 3.76)		4.85 (3.73, 5.98)		4.10 (2.76, 5.44)
Upper west		6.32 (5.06, 7.58)		2.81 (2.22, 3.40)		2.53 (1.85, 3.21)
Urbanicity	3183		2168		2388	
Urban		32.14 (29.78, 34.50)		36.95 (33.90, 40.00)		45.81 (41.73, 49.88)
Rural		67.86 (65.50, 70.22)		63.05 (60.00, 66.10)		54.19 (50.12, 58.27)
Child’s age (months)	3183		2168		2388	
6–11		21.56 (20.30, 22.81)		11.40 (9.83, 12.97)		11.28 (9.50, 13.06)
12–23		21.68 (20.00, 23.37)		23.82 (22.06, 25.59)		24.41 (22.57, 26.24)
24–35		19.45 (17.94, 20.96)		21.37 (19.35, 23.39)		22.59 (20.52, 24.65)
36–47		20.16 (18.74, 21.58)		20.66 (18.90, 22.42)		21.48 (19.53, 23.42)
48–59		17.15 (15.81, 18.49)		22.75 (20.87, 24.62)		20.25 (18.35, 22.14)
Sex	3183		2168		2388	
Male		50.55 (48.73, 52.36)		51.45 (49.11, 53.80)		52.95 (50.59, 55.31)
Female		49.45 (47.64, 51.27)		48.55 (46.20, 50.89)		47.05 (44.69, 49.41)
Child slept under net previous night	3183		2168		2388	
No		84.65 (82.56, 86.73)		56.05 (52.97, 59.14)		52.22 (48.84, 55.60)
Yes		15.35 (13.27, 17.44)		43.95 (40.86, 47.04)		47.78 (44.40, 51.16)
Had diarrhea recently	3172		2163		2388	
No		84.11 (82.63, 85.60)		77.59 (75.28, 79.81)		87.39 (85.46, 89.31)
Yes, last 2 weeks		15.89 (14.40, 17.37)		22.41 (20.19, 24.62)		12.62 (10.69, 14.54)
Had fever recently	3157		2162		2388	
No		77.99 (76.31, 79.68)		77.51 (75.28, 79.74)		84.72 (82.48, 86.95)
Yes, last 2 weeks		22.01 (20.32, 23.69)		22.49 (20.26, 24.72)		15.28 (13.05, 17.52)
Iron supplementation			2156		2379	
No				71.94 (69.40, 74.49)		76.77 (73.96, 79.59)
Yes				28.06 (25.51, 30.60)		23.23 (20.41, 26.04)
Drugs for parasites in last 6 mo			2151		2383	
No				57.23 (54.55, 59.91)		62.17 (59.18, 65.15)
Yes				42.77 (40.09, 45.45)		37.83 (34.85, 40.82)
Breastfeeding	3148		2159		2387	
Previously breastfed		56.58 (55.00, 58.16)		67.40 (65.37, 69.42)		68.00 (65.89, 70.10)
Never breastfed		0.37 (0.15, 0.58)		1.57 (0.91, 2.23)		1.03 (0.52, 1.54)
Still breastfeeding		43.06 (41.47, 44.64)		31.04 (29.06, 33.01)		30.98 (28.84, 33.11)
Dietary diversity (no. of food groups)			1684		1373	
< 4				64.75 (61.52, 67.98)		80.65 (77.41, 83.89)
≥ 4				35.25 (32.02, 38.48)		19.35 (16.11, 22.59)
Water source	3155		2156		2343	
Piped water		28.32 (24.85, 31.80)		33.76 (29.59, 37.93)		27.72 (24.17, 31.26)
Open well water		13.85 (11.29, 16.41)		36.84 (32.69, 40.99)		30.83 (26.35, 35.31)
Protected well water		31.42 (27.75, 35.10)		7.62 (5.12, 10.12)		10.10 (7.49, 12.70)
Surface water		25.63 (21.67, 29.58)		15.54 (11.89, 19.19)		10.70 (7.80, 13.61)
Bottled/sachet water		0.78 (0.32, 1.23)		6.24 (4.46, 8.01)		20.65 (17.47, 23.84)
Toilet	3156				2343	
Flush toilet		6.72 (4.85, 8.59)		8.48 (6.57, 10.40)		17.72 (14.67, 20.77)
Pit toilet latrine		62.05 (58.72, 65.37)		62.68 (58.75, 66.61)		56.84 (52.72, 60.95)
No facility		31.23 (28.10, 34.37)		28.69 (25.13, 32.54)		25.44 (21.64, 29.25)
Household wealth quintile	3183		2168		2388	
Poorest		26.50 (23.72, 29.29)		26.10 (22.61, 29.59)		22.82 (19.39, 26.25)
Poorer		22.51 (20.00, 25.01)		23.35 (20.55, 26.15)		20.47 (18.02, 22.92)
Middle		20.14 (17.71, 22.58)		17.63 (15.18, 20.08)		20.01 (17.18, 22.85)
Richer		16.50 (14.39, 18.60)		19.02 (16.55, 21.49)		18.34 (15.24, 21.44)
Richest		14.35 (12.05, 16.65)		13.90 (11.66, 16.13)		18.35 (15.09, 21.62)
Mother’s age (years)	3183		2168		2388	
15–19		3.54 (2.89, 4.18)		3.45 (2.56, 4.34)		2.74 (1.75, 3.73)
20–24		18.14 (16.28, 20.00)		18.62 (16.46, 20.78)		15.89 (13.88, 17.89)
25–29		26.38 (24.32, 28.44)		27.94 (25.31, 30.57)		24.31 (21.48, 27.15)
30–34		22.18 (20.25, 24.12)		20.38 (18.20, 22.55)		25.90 (23.25, 28.54)
35–39		17.35 (15.52, 19.18)		18.05 (15.98, 20.12)		19.65 (17.20, 22.11)
≥ 40		12.42 (10.91, 13.92)		11.56 (9.94, 13.18)		11.21 (9.78, 13.24)
Maternal education	3183		2168		2388	
No education		40.30 (37.45, 43.14)		33.18 (30.08, 36.28)		28.88 (25.23, 32.53)
Incomplete primary		16.53 (14.72, 18.34)		18.34 (16.09, 20.58)		13.85 (11.74, 15.97)
Complete primary		6.64 (5.45, 7.82)		6.28 (5.01, 7.55)		5.89 (4.59, 7.19)
Incomplete secondary		32.66 (30.44, 34.89)		35.23 (32.25, 38.20)		41.15 (37.48, 44.82)
Complete secondary		2.78 (1.90, 3.67)		4.63 (3.42, 5.85)		6.99 (5.40, 8.59)
Higher		1.09 (0.59, 1.60)		2.35 (1.48, 3.22)		3.24 (2.02, 4.45)
Health insurance coverage			2166		2388	
No				62.41 (59.22, 65.61)		33.25 (30.18, 36.33)
Yes				37.59 (34.39, 40.78)		66.75 (63.67, 69.82)
Maternal anemia type	3147		2150		2370	
Severe–moderate		9.38 (8.07, 10.69)		20.28 (17.88, 22.67)		9.81 (8.18, 11.44)
Mild		37.90 (35.70, 40.10)		41.31 (38.54, 44.08)		34.06 (31.33, 36.80)
Not anemic		52.72 (50.27, 55.17)		38.41 (35.37, 41.45)		56.13 (53.06, 59.19)
Number of children in household	3183		2168		2388	
1–2		38.72 (36.36, 41.08)		40.66 (37.65, 43.66)		42.94 (40.05, 45.82)
3		43.95 (41.60, 46.30)		41.81 (38.53, 45.09)		42.11 (39.04, 45.18)
≥ 4		17.33 (15.25, 19.41)		17.53 (14.62, 20.45)		14.95 (12.11, 17.80)
Number of other wives	3182		1965		2094	
0		71.02 (68.71, 73.33)		81.15 (78.34, 83.95)		84.96 (82.41, 87.51)
1		28.98 (26.67, 31.29)		18.86 (16.05, 21.66)		15.04 (12.49, 17.59)
Maternal decision-making autonomy	3134		1965		2108	
No autonomy		25.52 (22.94, 28.10)		10.27 (8.03, 12.52)		7.71 (5.61, 9.81)
Partial autonomy		34.95 (32.20, 37.70)		42.07 (38.84, 45.30)		34.70 (31.08, 38.33)
Full autonomy		39.53 (36.66, 42.40)		47.66 (43.86, 51.45)		57.59 (53.88, 61.29)
Maternal attitudes that justify beating	3182		2168		2388	
No		45.63 (42.93, 48.33)		40.98 (37.73, 44.23)		66.42 (62.48, 70.36)
Yes		54.37 (51.67, 57.07)		59.02 (55.77, 62.27)		33.58 (29.64, 37.52)
Religion	3181		2163		2388	
Christian		71.37 (68.68, 74.06)		71.29 (67.75, 74.84)		74.90 (71.13, 78.67)
Islam		17.83 (15.21, 20.45)		17.77 (14.08, 21.46)		18.20 (14.37, 22.03)
Traditional		4.29 (3.15, 5.43)		6.83 (4.51, 9.14)		3.39 (1.61, 5.18)
No religion		6.51 (4.94, 8.09)		4.11 (2.98, 5.24)		3.51 (2.47, 4.54)
Ethnicity	3181		2166		2388	
Akan		46.27 (42.98, 49.56)		47.10 (43.31, 50.89)		48.07 (43.30, 52.84)
Ga/Dangme		7.13 (5.35, 8.91)		4.59 (3.04, 6.15)		5.67 (3.89, 7.44)
Ewe		12.67 (10.89, 14.45)		12.43 (9.92, 14.94)		13.08 (10.51, 15.64)
Mole-Dagbani		16.67 (14.12, 19.21)		20.19 (16.64, 23.73)		17.02 (1337, 20.68)
Gurma		4.36 (2.54, 6.18)		5.49 (3.33, 7.65)		8.31 (5.22, 11.39)
Other		12.90 (10.62, 15.19)		10.20 (7.94, 12.47)		7.85 (5.94, 9.77)

Ghana Demographic and Health Survey, 2003–2014

### Prevalence and Distribution of Anemia

Severe–moderate anemia was found in 52%, 56%, and 40% of children during 2003, 2008, and 2014, respectively. The proportion of children diagnosed as not anemic was 26% in 2003, 22% in 2008, and 33% in 2014 (Tables 2, 3, 4). As compared to 2003, the prevalence of severe–moderate anemia increased slightly (4%) in 2008, but had substantially decreased (12%) by 2014. In 2014, 48% of children in rural areas had severe–moderate anemia compared to 30% in urban areas. While this significant disparity persisted through 2014, both urban and rural areas had a 10% drop in severe–moderate anemia as compared with 2003. severe–moderate anemia prevalence also differed by province across all survey years, ranging from 29% in Ashanti region (2014) to 62% in the Northern region (2014).

**Table 2:** Rao-Scott chi-square bivariate analysis of anemia type by sociodemographic factors for children 6–59 months from the 2014 Ghana Demographic and Health Survey (n = 2388)

	Severe–moderate anemia (weighted %)	Mild anemia (weighted %)	Non-anemic (weighted %)	P-value
Region				< 0.0001
Western	37.94	29.78	32.28	
Central	46.70	26.66	26.65	
Greater Accra	29.55	28.81	41.64	
Volta	43.45	27.25	29.30	
Eastern	38.52	28.00	33.48	
Ashanti	29.32	24.79	45.89	
Brong Ahafo	34.02	29.52	36.46	
Northern	61.57	22.93	15.50	
Upper east	43.95	30.61	25.44	
Upper west	52.28	21.36	26.36	
Urbanicity				< 0.0001
Urban	30.48	28.99	40.54	
Rural	47.69	25.31	27.00	
Child’s age (months)				< 0.0001
6–11	52.16	26.26	21.58	
12–23	52.25	24.61	23.14	
24–35	35.20	30.86	33.94	
36–47	33.77	27.44	38.79	
48–59	29.45	25.50	45.05	
Sex				0.8514
Male	39.21	27.50	33.30	
Female	40.47	26.44	33.09	
Child slept under net previous night				0.0404
No	36.70	27.97	35.33	
Yes	43.20	25.93	30.87	
Had diarrhea recently				0.0158
No	38.48	27.40	34.12	
Yes, last 2 weeks	49.00	24.19	26.81	
Had fever recently				< 0.0001
No	36.85	27.75	35.40	
Yes, last 2 weeks	56.15	22.84	21.02	
Iron supplementation				0.0015
No	42.09	25.23	32.67	
Yes	34.75	32.91	34.75	
Drugs for parasites in last 6 mo				< 0.0001
No	44.10	26.18	29.73	
Yes	32.69	28.36	38.95	
Breastfeeding				< 0.0001
Ever breastfed	32.97	28.38	38.65	
Never breastfed	43.89	17.84	38.26	
Still breastfeeding	54.74	24.14	21.11	
Dietary diversity (no. of food groups)				0.2195
< 4	48.75	25.11	26.14	
≥ 4	40.72	28.44	30.84	
Water source				< 0.0001
Piped water	35.44	27.07	37.49	
Tube well water	49.98	23.38	26.64	
Dug well	50.21	23.85	25.94	
Surface water	52.08	25.79	22.13	
Bottled/sachet water	20.74	32.79	46.46	
Toilet				< 0.0001
Flush toilet	18.36	34.19	47.46	
Pit toilet latrine	40.32	24.84	34.84	
No facility	54.98	25.45	19.57	
Wealth				< 0.0001
Poorest	54.96	25.81	19.23	
Poorer	51.02	25.60	23.38	
Middle	43.21	20.11	36.69	
Richer	26.47	33.64	39.89	
Richest	18.05	30.91	51.04	
Mother’s age (years)				0.1247
15–19	59.09	18.41	22.50	
20–24	46.00	26.88	27.12	
25–29	39.63	26.09	34.28	
30–34	36.21	27.14	36.64	
35–39	37.78	27.84	34.38	
≥ 40	38.54	29.35	32.11	
Maternal education				< 0.0001
No education	54.20	25.93	19.87	
Incomplete primary	41.57	27.42	31.01	
Complete primary	48.48	18.32	33.20	
Incomplete secondary	32.74	27.81	39.45	
Complete secondary	24.54	30.31	45.15	
Higher	10.76	33.03	56.21	
Covered by health insurance				0.0006
No	46.57	25.80	27.63	
Yes	36.43	27.59	35.97	
Mother’s anemia level				< 0.0001
Moderate–severe	53.76	27.69	18.55	
Mild	45.40	25.59	29.00	
Not anemic	34.06	27.82	38.12	
Number of children in household				< 0.0001
1–2	32.58	28.39	39.03	
3	44.15	25.66	30.19	
≥ 4	48.31	26.77	24.92	
Number of other wives				< 0.0001
0	37.60	26.51	35.89	
≥ 1	53.05	27.26	37.60	
Maternal decision-making autonomy				0.0336
No autonomy	51.50	25.86	22.64	
Partial autonomy	41.57	27.17	31.27	
Full autonomy	37.24	26.79	35.97	
Maternal attitudes that justify beating				< 0.0001
No	34.27	23.75	25.51	
Yes	50.75	28.64	37.09	
Religion				< 0.0001
Christian	36.23	27.21	36.56	
Islam	46.92	27.04	26.04	
Traditional	72.76	15.85	11.39	
No religion	47.22	33.00	19.78	
Ethnicity				< 0.0001
Akan	34.76	25.83	39.41	
Ga/Dangme	37.51	29.30	33.20	
Ewe	36.15	30.29	33.56	
Mole-Dagbani	45.83	26.50	27.67	
Gurma	59.39	26.15	14.46	
Other	44.61	29.00	26.39	

P-values show whether there is a statistically significant difference between categories of each variable

**Table 3:** Rao-Scott chi-square bivariate analysis of anemia type by sociodemographic factors for children 6–59 months from the 2008 Ghana Demographic and Health Survey (n = 2168)

	Severe–moderate anemia (weighted %)	Mild anemia (weighted %)	Non anemic (weighted %)	P-value
Region				< 0.0001
Western	62.03	17.36	20.61	
Central	62.55	22.89	14.57	
Greater Accra	30.99	31.33	37.68	
Volta	60.26	19.21	20.53	
Eastern	48.28	25.11	26.61	
Ashanti	56.38	23.39	20.23	
Brong Ahafo	54.12	23.91	21.98	
Northern	65.47	16.54	17.99	
Upper east	58.33	30.62	11.05	
Upper west	74.51	13.55	11.94	
Urbanicity				< 0.0001
Urban	43.29	24.29	32.41	
Rural	63.15	21.62	15.23	
Child’s age (months)				< 0.0001
6–11	67.34	17.89	14.77	
12–23	64.33	20.76	14.90	
24–35	57.61	22.40	19.98	
36–47	48.47	25.57	25.96	
48–59	46.08	24.41	29.52	
Sex				0.4260
Male	56.77	22.86	20.37	
Female	54.80	22.34	22.86	
Child slept under net previous night				0.1338
No	54.02	22.78	23.19	
Yes	58.09	22.38	19.53	
Had diarrhea recently				< 0.0001
No	53.12	23.16	23.72	
Yes, last 2 weeks	65.26	20.55	14.19	
Had fever recently				0.0015
No	53.80	22.84	23.36	
Yes, last 2 weeks	62.49	22.00	15.51	
Iron supplementation				0.6630
No	55.80	23.08	21.12	
Yes	55.70	21.48	22.82	
Drugs for parasites in last 6 mo				0.0021
No	58.91	22.32	18.77	
Yes	51.83	23.02	25.15	
Breastfeeding				< 0.0001
Ever breastfed	50.70	23.98	25.32	
Never breastfed	40.41	24.99	34.60	
Still breastfeeding	67.44	19.82	12.74	
Dietary diversity (no. of food groups)				0.9970
< 4	59.61	21.46	18.92	
≥ 4	59.50	21.40	19.10	
Water source				< 0.0001
Piped water	48.52	24.99	26.49	
Tube well water	62.51	22.21	15.28	
Dug well	54.07	24.49	21.44	
Surface water	66.68	17.05	16.27	
Bottled/sachet water	30.01	23.87	46.11	
Toilet				< 0.0001
Flush toilet	28.56	28.91	42.52	
Pit toilet latrine	54.53	23.52	21.94	
No facility	66.38	18.74	14.88	
Wealth				< 0.0001
Poorest	66.84	20.35	12.81	
Poorer	61.55	22.28	16.17	
Middle	60.53	20.33	19.14	
Richer	48.57	21.85	29.57	
Richest	29.36	31.31	39.32	
Mother’s age (years)				0.0198
15–19	68.88	19.98	11.14	
20–24	59.76	23.77	16.46	
25–29	57.89	21.11	21.01	
30–34	48.43	25.65	25.93	
35–39	54.61	22.35	23.04	
≥ 40	55.42	20.18	24.41	
Maternal education				< 0.0001
No education	64.95	18.45	16.60	
Incomplete primary	58.58	22.56	18.87	
Complete primary	53.48	26.02	20.50	
Incomplete secondary	50.85	25.40	23.75	
Complete secondary	36.16	23.24	40.60	
Higher	24.52	29.40	46.08	
Covered by health insurance				< 0.0001
No	59.79	21.39	18.82	
Yes	49.12	24.67	26.21	
Mother’s anemia level				< 0.0001
Moderate–severe	69.23	17.80	12.97	
Mild	56.79	22.41	20.80	
Not anemic	47.44	25.48	27.08	
Number of children in household				0.1567
1–2	53.88	22.85	23.28	
3	55.17	23.01	21.82	
≥ 4	61.82	21.10	17.08	
Number of other wives				0.0023
0	54.23	22.86	22.91	
≥ 1	64.73	19.40	15.87	
Maternal decision-making autonomy				0.0489
No autonomy	65.03	20.87	14.10	
Partial autonomy	56.70	21.19	22.11	
Full autonomy	53.89	23.68	22.43	
Maternal attitudes that justify beating				< 0.0001
No	50.71	25.29	24.00	
Yes	63.16	18.74	18.10	
Religion				0.0008
Christian	52.88	23.78	23.33	
Islam	62.28	17.92	19.81	
Traditional	66.75	20.64	12.61	
No religion	61.40	24.93	13.68	
Ethnicity				0.0010
Akan	52.56	23.61	23.83	
Ga/Dangme	43.75	26.47	29.78	
Ewe	55.67	24.10	20.23	
Mole-Dagbani	60.98	19.96	19.07	
Gurma	74.56	17.16	8.27	
Other	55.85	22.72	21.42	

P-values show whether there is a statistically significant difference between categories of each variable

**Table 4:** Rao-Scott chi-square bivariate analysis of anemia type by sociodemographic factors for children 6–59 months from the 2003 Ghana Demographic and Health Survey (n = 3183)

	Severe–moderate anemia (weighted %)	Mild anemia (weighted %)	Non anemic (weighted %)	P-value
Region				< 0.0001
Western	56.33	22.33	21.34	
Central	52.54	23.85	23.60	
Greater Accra	37.33	23.32	39.35	
Volta	48.52	24.76	26.71	
Eastern	48.26	20.67	21.07	
Ashanti	55.34	21.91	22.75	
Brong Ahafo	50.44	21.39	28.18	
Northern	60.12	19.01	20.87	
Upper east	51.50	22.49	26.01	
Upper west	57.30	22.83	19.87	
Urbanicity				< 0.0001
Urban	40.94	25.27	33.79	
Rural	57.40	20.47	22.12	
Child’s age (months)				< 0.0001
6–11	47.39	18.52	34.09	
12–23	64.60	19.12	16.28	
24–35	55.75	21.59	22.65	
36–47	49.91	24.17	25.92	
48–59	40.72	28.01	31.27	
Sex				0.9986
Male	52.06	22.04	25.90	
Female	52.16	21.99	25.84	
Child slept under net previous night				0.3841
No	52.64	21.90	25.47	
Yes	49.21	22.68	28.11	
Had diarrhea recently				0.0043
No	50.85	22.09	27.06	
Yes, last 2 weeks	58.66	21.46	19.88	
Had fever recently				< 0.0001
No	49.58	22.60	27.82	
Yes, last 2 weeks	60.56	20.49	18.95	
Iron supplementation				
No				
Yes				
Drugs for parasites in last 6 mo				
No				
Yes				
Breastfeeding				0.0002
Ever breastfed	48.53	24.69	26.78	
Never breastfed	34.67	26.55	38.78	
Still breastfeeding	57.16	18.54	24.29	
Dietary diversity (no. of food groups)				
< 4				
≥ 4				
Water source				< 0.0001
Piped water	41.10	24.24	34.66	
Open well water	56.11	24.73	19.17	
Protected well water	56.78	19.90	23.31	
Surface water	56.77	20.21	23.02	
Bottled/sachet water	45.98	30.72	23.30	
Toilet				< 0.0001
Flush toilet	30.92	32.23	36.84	
Pit toilet latrine	51.36	21.70	26.94	
No facility	58.02	20.42	21.56	
Wealth				< 0.0001
Poorest	61.72	18.77	19.50	
Poorer	56.41	21.85	21.73	
Middle	54.38	22.50	23.13	
Richer	44.76	22.13	33.11	
Richest	32.89	27.45	39.66	
Mother’s age (years)				0.0689
15–19	49.48	25.07	25.45	
20–24	58.67	17.09	24.23	
25–29	52.53	22.53	24.93	
30–34	47.66	25.69	25.65	
35–39	51.66	21.74	26.60	
≥ 40	50.97	21.06	27.97	
Maternal education				< 0.0001
No education	58.74	20.20	21.05	
Incomplete primary	54.06	22.07	23.87	
Complete primary	54.49	24.28	21.23	
Incomplete secondary	44.53	23.34	32.13	
Complete secondary	33.29	29.19	37.52	
Higher	38.34	16.58	45.08	
Covered by health insurance				
No				
Yes				
Mother’s anemia level				0.0013
Moderate–severe	48.65	22.80	28.55	
Mild	55.99	20.63	23.38	
Not anemic	56.33	23.82	19.85	
Number of children in household				0.0030
1–2	47.59	23.71	28.71	
3	53.93	21.77	24.31	
≥ 4	57.62	18.87	23.51	
Number of other wives				< 0.0001
0	49.67	22.25	28.08	
≥ 1	58.23	21.51	20.26	
Maternal decision-making autonomy				0.0093
No autonomy	54.43	21.77	23.80	
Partial autonomy	54.58	18.97	26.46	
Full autonomy	48.60	24.93	26.46	
Maternal attitudes that justify beating				0.0033
No	54.58	22.15	23.27	
Yes	49.21	21.87	28.91	
Religion				0.0547
Christian	50.52	22.16	27.32	
Islam	54.79	22.15	23.06	
Traditional	61.99	20.40	17.61	
No religion	55.85	20.69	23.47	
Ethnicity				0.0381
Akan	50.82	22.35	26.83	
Ga/Dangme	47.49	21.38	31.12	
Ewe	48.96	22.35	28.70	
Mole-Dagbani	56.66	22.65	20.69	
Gurma	62.98	15.94	21.08	
Other	52.86	21.82	25.32	

P-values show whether there is a statistically significant difference between categories of each variabl

### Factors Associated with Mild Anemia

In the 2014 multivariable logistic regression model, few factors were significantly associated with mild anemia (Table 5). Children aged 48–59 months were less likely to be anemic (OR 0.49; 95% CI 0.31, 0.79) than children aged 12–23 months. Children of mothers with severe–moderate anemia were more likely to be anemic (OR 2.04, 95% CI 1.13, 3.66) than children whose mothers did not have severe–moderate anemia. In 2008, no factors examined were significantly associated with mild anemia. In 2003, child’s age (OR 0.43, 95% CI 0.30, 0.64) and mother’s anemia status (OR 1.65, 95% CI 1.11, 2.45) were associated with child’s anemia status; in addition, using open well water versus piped water was associated with higher odds of mild anemia (OR 1.62, 95% CI 1.03, 2.55).

**Table 5:** Adjusted odds ratios for anemia type by sociodemographic covariates for children 6–59 months from the 2003, 2008, and 2014 Ghana Demographic and Health Surveys

Covariates	2003 (n = 3028)		2008 (n = 1888)		2014 (n = 2030)	
	Severe–moderate anemia versus non-anemic	Mild anemia versus non-anemic	Severe–moderate anemia versus non-anemic	Mild anemia versus non-anemic	Severe–moderate anemia versus non-anemic	Mild anemia versus non-anemic
Urbanicity						
Urban	Ref	Ref	Ref	Ref	Ref	Ref
Rural	1.12 (0.76, 1.66)	0.96 (0.62, 1.48)	1.61 (1.05, 2.47)	1.37 (0.88, 2.13)	0.84 (0.55, 1.27)	0.92 (0.60, 1.41)
Child’s age (months)						
6–11	**0.31 (0.22, 0.42)**	**0.43 (0.30, 0.64)**	0.90 (0.52, 1.58)	0.84 (0.44, 1.61)	1.39 (0.81, 2.38)	1.43 (0.82, 2.49)
12–23	Ref	Ref	Ref	Ref	Ref	Ref
24–35	**0.61 (0.44, 0.87)**	0.83 (0.57, 1.21)	**0.62 (0.39, 1.00)**	0.71 (0.41, 1.21)	**0.48 (0.32, 0.73)**	1.06 (0.69, 1.63)
36–47	**0.46 (0.33, 0.66)**	0.83 (0.55, 1.23)	**0.40 (0.25, 0.64)**	0.67 (0.39, 1.16)	**0.32 (0.21, 0.48)**	**0.64 (0.42, 0.98)**
48–59	**0.32 (0.22, 0.45)**	0.80 (0.56, 1.14)	**0.34 (0.22, 0.55)**	**0.54 (0.32, 0.93)**	**0.20 (0.13, 0.31)**	**0.49 (0.31, 0.79)**
Sex						
Male	Ref	Ref	Ref	Ref	Ref	Ref
Female	1.07 (0.86, 1.32)	1.07 (0.84, 1.36)	0.84 (0.65, 1.08)	0.81 (0.60, 1.11)	0.84 (0.62, 1.14)	0.86 (0.61, 1.21)
Child slept under net previous night						
No	Ref	Ref	Ref	Ref	Ref	Ref
Yes	0.79 (0.59, 1.06)	0.96 (0.70, 1.31)	1.11 (0.83, 1.50)	1.01 (0.72, 1.42)	1.03 (0.75, 1.42)	0.94 (0.70, 1.28)
Had diarrhea recently						
No	Ref	Ref	Ref	Ref	Ref	Ref
Yes, last 2 weeks	1.14 (0.83, 1.55)	1.15 (0.79, 1.67)	1.37 (0.93, 2.02)	1.37 (0.87, 2.15)	1.13 (0.68, 1.85)	1.01 (0.60, 1.70)
Had fever recently						
No	Ref	Ref	Ref	Ref	Ref	Ref
Yes, last 2 weeks	**1.60 (1.20, 2.13)**	1.20 (0.88, 1.63)	**1.59 (1.10, 2.29)**	1.36 (0.88, 2.09)	**2.39 (1.58, 3.63)**	1.32 (0.87, 2.01)
Iron supplementation						
No			Ref	Ref	Ref	Ref
Yes			0.94 (0.67, 1.33)	0.86 (0.57, 1.30)	0.76 (0.53, 1.08)	1.30 (0.89, 1.88)
Drugs for parasites in last 6 mo						
No			Ref	Ref	Ref	Ref
Yes			1.01 (0.72, 1.42)	1.01 (0.71, 1.43)	1.05 (0.73, 1.49)	1.11 (0.79, 1.55)
Water source						
Piped water	Ref	Ref	Ref	Ref	Ref	Ref
Open well water	1.34 (0.87, 2.06)	**1.62 (1.03, 2.55)**	1.13 (0.71, 1.80)	1.28 (0.77, 2.15)	1.25 (0.78, 2.01)	1.08 (0.69, 1.69)
Protected well water	1.10 (0.73, 1.64)	1.08 (0.68, 1.71)	1.12 (0.58, 2.17)	1.60 (0.88, 2.89)	1.31 (0.67, 2.55)	1.08 (0.58, 2.01)
Surface water	1.07 (0.68, 1.68)	1.12 (0.67, 1.88)	0.99 (0.56, 1.76)	0.84 (0.43, 1.64)	1.19 (0.66, 2.13)	1.23 (0.64, 2.35)
Bottled/sachet water	2.30 (0.58, 9.12)	2.10 (0.53, 8.31)	0.59 (0.31, 1.14)	0.50 (0.23, 1.10)	0.91 (0.54, 1.55)	1.41 (0.90, 2.21)
Toilet						
Flush toilet	Ref	Ref	Ref	Ref	Ref	Ref
Pit toilet latrine	1.07 (0.67, 1.72)	**0.69 (0.48, 0.99)**	1.47 (0.81, 2.67)	1.30 (0.71, 2.40)	1.51 (0.84, 2.70)	0.75 (0.46, 1.21)
No facility	1.01 (0.60, 1.71)	0.69 (0.45, 1.07)	1.73 (0.82, 3.63)	1.12 (0.52, 2.45)	1.93 (0.99, 3.77)	1.10 (0.62, 1.95)
Wealth						
Poorest	Ref	Ref	Ref	Ref	Ref	Ref
Poorer	0.96 (0.65, 1.40)	1.24 (0.85, 1.81)	1.13 (0.69, 1.87)	0.98 (0.55, 1.75)	1.14 (0.72, 1.81)	1.11 (0.65, 1.89)
Middle	0.91 (0.60, 1.39)	1.27 (0.82, 1.99)	1.05 (0.55, 2.01)	0.67 (0.32, 1.41)	0.67 (0.38, 1.18)	**0.52 (0.28, 0.96)**
Richer	0.59 (0.34, 1.02)	0.82 (0.45, 1.48)	0.70 (0.34, 1.43)	0.63 (0.30, 1.34)	0.48 (0.22, 1.05)	0.90 (0.43, 1.89)
Richest	**0.42 (0.22, 0.81)**	0.87 (0.46, 1.65)	0.58 (0.24, 1.40)	1.04 (0.41, 2.65)	**0.33 (0.14, 0.74)**	0.45 (0.19, 1.09)
Mother’s age (years)						
15–19	0.69 (0.38, 1.26)	1.14 (0.57, 2.30)	1.05 (0.35, 3.22)	1.14 (0.31, 4.17)	4.95 (1.17, 21.13)	3.18 (0.71, 14.22)
20–24	Ref	Ref	Ref	Ref	Ref	Ref
25–29	0.90 (0.65, 1.26)	1.30 (0.91, 1.87)	0.94 (0.60, 1.48)	0.78 (0.46, 1.32)	0.86 (0.50, 1.47)	0.77 (0.41, 1.42)
30–34	0.79 (0.57, 1.09)	1.33 (0.92, 1.92)	0.76 (0.48, 1.22)	0.77 (0.43, 1.40)	1.03 (0.63, 1.69)	0.99 (0.57, 1.72)
35–39	**0.68 (0.48, 0.98)**	0.97 (0.64, 1.45)	0.95 (0.54, 1.66)	0.76 (0.39, 1.49)	1.08 (0.62, 1.87)	0.97 (0.56, 1.70)
≥ 40	0.70 (0.47, 1.05)	0.86 (0.53, 1.39)	0.75 (0.45, 1.26)	0.72 (0.37, 1.38)	0.70 (0.40, 1.22)	0.89 (0.47, 1.71)
Maternal education						
No education	Ref	Ref	Ref	Ref	Ref	Ref
Primary (complete or incomplete)	0.87 (0.63, 1.22)	1.03 (0.74, 1.44)	0.87 (0.56, 1.36)	1.30 (0.81, 2.09)	0.67 (0.44, 1.03)	0.74 (0.47, 1.16)
Secondary or higher	**0.58 (0.42, 0.79)**	0.80 (0.57, 1.11)	0.92 (0.57, 1.49)	1.36 (0.80, 2.32)	**0.53 (0.34, 0.81)**	0.81 (0.51, 1.27)
Covered by health insurance						
No			Ref	Ref	Ref	Ref
Yes			**0.69 (0.51, 0.93)**	0.82 (0.57, 1.17)	0.74 (0.53, 1.04)	0.97 (0.70, 1.36)
Mother’s Anemia Level						
Moderate–severe	**1.73 (1.19, 2.51)**	**1.65 (1.11, 2.45)**	**2.89 (1.90, 4.38)**	1.30 (0.81, 2.10)	**2.66 (1.46, 4.83)**	**2.04 (1.13, 3.66)**
Mild	**1.35 (1.10, 1.66)**	1.11 (0.85, 1.44)	1.345 (0.972, 1.86)	1.15 (0.78, 1.69)	**1.80 (1.28, 2.54)**	1.28 (0.87, 1.89)
Not anemic	Ref	Ref	Ref	Ref	Ref	Ref
Number of children in household						
1–2	Ref	Ref	Ref	Ref	Ref	Ref
3	**1.26 (1.02, 1.57)**	1.02 (0.80, 1.30)	0.99 (0.73, 1.34)	1.07 (0.75, 1.528)	1.32 (0.93, 1.87)	1.24 (0.87, 1.76)
≥ 4	1.21 (0.89, 1.64)	0.87 (0.62, 1.22)	1.04 (0.68, 1.59)	1.05 (0.65, 1.689)	1.57 (0.97, 2.56)	1.51 (0.94, 2.44)
Number of other wives						
0	Ref	Ref	Ref	Ref	Ref	Ref
≥ 1	**1.36 (1.07, 1.71)**	**1.36 (1.05, 1.76)**	1.13 (0.75, 1.70)	1.10 (0.69, 1.748)	1.22 (0.78, 1.91)	1.21 (0.73, 2.00)
Maternal decision-making autonomy						
No autonomy	Ref	Ref	Ref	Ref	Ref	Ref
Partial autonomy	0.94 (0.67, 1.25)	0.78 (0.56, 1.07)	0.53 (0.31, 0.90)	0.60 (0.35. 1.008)	1.02 (0.50, 2.05)	0.84 (0.42, 1.68)
Full autonomy	0.90 (0.67, 1.22)	1.06 (0.77, 1.45)	0.56 (0.32, 0.98)	0.67 (0.38, 1.206)	0.69 (0.36, 1.32)	0.81 (0.41, 1.60)
Maternal attitudes that justify beating						
Yes	Ref	Ref	Ref	Ref	Ref	Ref
No	0.97 (0.77, 1.22)	0.90 (0.70, 1.16)	1.00 (0.73, 1.37)	1.41 (1.00, 2.00)	0.81 (0.58, 1.12)	1.01 (0.74, 1.36)
Religion						
Christian	Ref	Ref	Ref	Ref	Ref	Ref
Islam	1.03 (0.74, 1.42)	1.02 (0.70, 1.49)	1.14 (0.67, 1.93)	0.78 (0.42, 1.450)	1.35 (0.81, 2.24)	1.19 (0.75, 1.86)
Traditional	1.17 (0.74, 1.86)	1.28 (0.71, 2.29)	1.14 (0.61, 2.13)	1.19 (0.56, 2.503)	2.02 (0.74, 5.50)	1.01 (0.31, 3.22)
No religion	0.89 (0.62, 1.28)	1.09 (0.67, 1.75)	1.14 (0.51, 2.55)	1.43 (0.62, 3.306)	1.91 (0.81, 4.51)	**3.04 (1.33, 6.93)**
Ethnicity						
Akan	Ref	Ref	Ref	Ref	Ref	Ref
Ga/Dangme	0.80 (0.49, 1.33)	0.80 (0.48, 1.34)	0.96 (0.48, 1.94)	1.41 (0.78, 2.526)	**1.92 (1.05, 3.50)**	1.27 (0.67, 2.39)
Ewe	0.77 (0.55, 1.08)	0.87 (0.60, 1.27)	1.20 (0.71, 2.00)	1.41 (0.80, 2.467)	0.88 (0.52, 1.47)	1.15 (0.72, 1.81)
Mole-Dagbani	0.88 (0.58, 1.33)	1.20 (0.78, 1.86)	0.95 (0.55, 1.62)	1.45 (0.79, 2.656)	0.62 (0.34, 1.12)	0.95 (0.53, 1.70)
Gurma	0.90 (0.51, 1.58)	0.93 (0.50, 1.75)	**2.05 (1.04, 4.05)**	1.73 (0.75, 3.971)	1.10 (0.42, 2.86)	1.50 (0.65, 3.46)
Other	0.77 (0.50, 1.18)	0.96 (0.60, 1.55)	0.93 (0.52, 1.66)	1.36 (0.72, 2.565)	0.87 (0.47, 1.60)	1.30 (0.70, 2.40)

Bolded values are significant at the < 0.05 level

### Factors Associated with Severe–Moderate Anemia

In 2014, child’s age had a strong association with severe–moderate anemia. As children aged, their odds of anemia became progressively lower; as compared to children 12–23 months old, those aged 24–35 months had 0.48 (95% CI 0.32, 0.73) times the odds of anemia, and others aged 48–59 months had 0.20 (95% CI 0.13, 0.31) times the odds of anemia. Fever in the previous 2 weeks was significantly associated with increased odds of severe–moderate anemia (OR 2.39, 95% CI 1.58, 3.63).

The other variables significantly associated with childhood severe–moderate anemia in 2014 were directly related to maternal characteristics or household factors. Mothers who had secondary or higher education were less likely to have moderately to severely anemic children compared to uneducated mothers (OR 0.53, 95% CI 0.34, 0.81). Children were significantly more likely to be moderately-severely anemic when their mothers had mild anemia (OR 1.80, 95% CI 1.28, 2.54) or severe–moderate anemia (OR 2.66, 95% CI 1.46, 4.83). Lastly, children in the richest wealth quintile had 0.33 (95% CI 0.14, 0.74) times the odds of severe–moderate anemia compared to children in the poorest wealth quintile (Table 5).

In 2008, child’s age, fever in the previous 2 weeks, and maternal anemia status were significantly associated with childhood severe–moderate anemia, similar to 2014. However, household wealth (OR 0.92, 95% CI 0.57, 1.49) and maternal education (OR 0.58, 95% CI 0.24, 1.40) were not associated with childhood severe–moderate anemia. In 2008, unlike in 2014, children were significantly less likely to have severe–moderate anemia if their mother's decision-making autonomy was partial (OR 0.53, 95% CI: 0.31, 0.90) or full (OR 0.56, 95% CI 0.32, 0.98). Furthermore, those children in families with health insurance had lower odds of anemia compared to other children without access to health insurance (OR 0.69, 95% CI 0.51, 0.93).

In 2003, child’s age was significantly associated with severe–moderate anemia, similar to 2008 and 2014. As in 2014, but unlike 2008, household wealth and maternal education did have a significant association with severe–moderate anemia during childhood in 2003.

## Discussion

Anemia prevalence in Ghana is exceptionally high. Globally, anemia prevalence in young children (6–59 months old) is estimated at 43% as compared with 67% in Ghana (Stevens et al. 2013). From our analysis, ~ 40% of Ghanaian children aged 6–59 months had severe–moderate anemia in 2014. Although this represents a substantial improvement over 2003 and 2008, there is evidence of widening disparities across regions of Ghana. In 2003, severe–moderate anemia prevalence difference was 23% between the highest (Northern region) and the lowest (Greater Accra region). However, that gap increased to 34% in 2008 and 32% in 2014. There was also an urban–rural disparity in severe–moderate anemia prevalence (17% higher in rural areas in 2014), but this disparity has remained relatively constant over time.

Across all three survey years, child’s age was negatively associated with severe–moderate anemia, as older children had lower odds of anemia than younger children. The high prevalence of severe–moderate anemia in children under 2 years old appears related to poor maternal nutrition, as children born to malnourished mothers have poor stores of many essential micronutrients, such as iron, zinc, vitamin A and B_12_ and folate (Kotecha 2011; Neumann et al. 2004). Our finding that children with mothers who have anemia have higher odds of developing anemia themselves, provides further evidence for this (Table 5).

Complementary foods and feeding practices are especially important for determining the micronutrient adequacy of 6–23 month-old children, as breast milk makes a progressively smaller contribution to an infant’s nutritional requirements into late infancy and early childhood (Saaka and Galaa 2017; Woldie et al. 2015). Our findings are consistent with this observation, in that the prevalence of severe–moderate anemia was higher among children who were still being breastfed than those who never or formerly breastfed (Tables 2, 3, 4). This likely reflects inadequate consumption of high quality complementary foods with continued breastfeeding rather than a deleterious effect of continued breastfeeding itself on iron status or anemia (Cumber et al. 2017; Lander et al. 2009). Reverse causality may also underlie this association (i.e., smaller, malnourished children may be weaned later because of perceived vulnerability) (Habicht 2000; Simondon et al. 2001).

Infants also generally have a higher incidence of infectious diseases, which can reduce their ability to ingest and absorb iron, perhaps further explaining the higher prevalence of anemia among younger children (Villalpando et al. 2003). From bivariate analyses across all years, we determined that a higher prevalence of anemia was associated with diarrhea or fever experienced within the previous 2 weeks (Tables 2, 3, 4). Although diarrhea and fever were associated with anemia, the cross-sectional nature of observations precludes determining the direction of the association. Participant-reported diarrhea or fever was asked for a period preceding the Hb measurement, although we cannot determine the temporal pattern of any association. One published prospective study has suggested that anemia may be a precursor of diarrheal disease (Levy et al. 2005). The relationship could also be cyclic, with diarrhea initially decreasing the body's ability to ingest and absorb iron, hence increasing subsequent risk of anemia, and anemia then increasing the risk of additional diarrheal episodes (Semba et al. 2008).

The association between fever in the past 2 weeks and severe–moderate anemia persisted in the multivariable model for all survey years, however, this was not the case for diarrheal illness. In our model, we controlled for both water source and toilet type (often a proxy for sanitation), which could have attenuated any association between diarrheal illness and anemia, as cleaner water and improved sanitation are associated with a lower risk of diarrheal pathogen exposure and illness (WHO 2014). More broadly, socioeconomic status (e.g. household wealth and education) also has been shown to predict health outcomes (Cameron and Williams 2009; Feinstein 1993). We included both household wealth and education in our multivariable models, which could have controlled for the association between two health indicators, such as diarrhea and anemia (see Fig. 1).

In 2003 and 2014, children from higher-income households and children with more highly educated mothers had lower odds of severe–moderate anemia in multivariable analysis. (For 2008, these associations were only significant in bivariate analysis.) It seems reasonable that households with higher incomes are better equipped to keep their children healthy due to a greater ability to purchase health services and food with higher nutritional value. Additionally, highly educated mothers are more likely to be knowledgeable about how to care for their own health, and that of their child.

### Limitations and Strengths

Our findings should be interpreted within the context of several limitations. The cross-sectional nature of the GDHS allows us to look for statistical associations, but not to assess temporality or causality. Survivor bias is also a possible issue; we only included surviving children and deaths could have resulted from complications due to anemia. Although this study analyzed data from three rounds of the GDHS, and each year's survey was similar, some variables such as health insurance coverage, iron supplementation, and parasites treatment were only available in later datasets, requiring slightly different multivariable models by year. Additionally, all datasets lacked information on factors such as malaria infection, so we were unable to consider the impact they had on anemia.

This study also has many strengths. The large sample size helped avoid small cell sizes in the multivariable analysis, allowing us to test multiple associations between potential predictors and anemia status with adequate statistical power. To the authors knowledge, this is the first study to assess predictors of anemia in Ghana over three different rounds of GDHS data.

## Conclusions

The prevalence of anemia among children aged 6–59 months in 2014 remains unacceptably high, even though substantial improvement has occurred since 2003. Despite overall advances, Ghana has experienced widening disparities in the prevalence of anemia among regions, and a persistent disparity between urban and rural areas. Results demonstrate that younger child age, fever in the last 2 weeks, lower household wealth, less maternal education, and maternal anemia are significantly associated with greater severe–moderate anemia. Associations have remained relatively consistent over the 2003, 2008 and 2014 time periods. In order to decrease anemia prevalence among children, the Ghanaian government should concentrate on educating women, both through the traditional education system and through antenatal care visits. Antenatal care visits could also provide an opportunity to ensure mothers are receiving adequate nutrition during pregnancy, as this has significant implications for the amount of iron available to infants through breastfeeding. A focus on improving water sources and sanitation in Ghana would also be beneficial, since they are often seen as predictive of diarrheal disease, which may have a cyclic effect on anemia.
